# Cold sensitivity and associated factors: a nested case–control study performed in Northern Sweden

**DOI:** 10.1007/s00420-018-1327-2

**Published:** 2018-05-28

**Authors:** Albin Stjernbrandt, Daniel Carlsson, Hans Pettersson, Ingrid Liljelind, Tohr Nilsson, Jens Wahlström

**Affiliations:** 1 Department of Public Health and Clinical Medicine, Occupational and Environmental Medicine, Umeå University, 901 87 Umeå, Sweden; 2 Occupational and Environmental Medicine, University Hospital of Umeå, 901 85 Umeå, Sweden

**Keywords:** Cold exposure, Sweden, Hand, Frostbite, Cold sensitivity

## Abstract

**Purpose:**

To identify factors associated with the reporting of cold sensitivity, by comparing cases to controls with regard to anthropometry, previous illnesses and injuries, as well as external exposures such as hand–arm vibration (HAV) and ambient cold.

**Methods:**

Through a questionnaire responded to by the general population, ages 18–70, living in Northern Sweden (*N* = 12,627), cold sensitivity cases (*N* = 502) and matched controls (*N* = 1004) were identified, and asked to respond to a second questionnaire focusing on different aspects of cold sensitivity as well as individual and external exposure factors suggested to be related to the condition. Conditional logistic regression analyses were performed to determine statistical significance.

**Results:**

In total, 997 out of 1506 study subjects answered the second questionnaire, yielding a response rate of 81.7%. In the multiple conditional logistic regression model, identified associated factors among cold sensitive cases were: frostbite affecting the hands (OR 10.3, 95% CI 5.5–19.3); rheumatic disease (OR 3.1, 95% CI 1.7–5.7); upper extremity nerve injury (OR 2.0, 95% CI 1.3–3.0); migraines (OR 2.4, 95% CI 1.3–4.3); and vascular disease (OR 1.9, 95% CI 1.2–2.9). A body mass index ≥ 25 was inversely related to reporting of cold sensitivity (0.4, 95% CI 0.3–0.6).

**Conclusions:**

Cold sensitivity was associated with both individual and external exposure factors. Being overweight was associated with a lower occurrence of cold sensitivity; and among the acquired conditions, both cold injuries, rheumatic diseases, nerve injuries, migraines and vascular diseases were associated with the reporting of cold sensitivity.

## Introduction

Cold sensitivity is an elusive condition that has previously been defined as an exaggerated or abnormal reaction to cold exposure, causing discomfort or the avoidance of cold (Kay 1985). It can be accompanied by pain, numbness, stiffness, weakness, swelling and skin color changes in the affected body part, most often the hands (Irwin et al. 1997). However, there is no universally accepted symptom-based definition of cold sensitivity, although attempts have been made (Lithell et al. 1997). The pathophysiological mechanisms are not fully elucidated, but seem to involve a multifactorial etiology, including neural (Irwin et al. 1997), vascular (Hope et al. 2014), as well as humoral (Koman et al. 1998) aspects. Cold sensitivity has previously been studied as a sequela to upper extremity injuries, such as digital and hand amputation (Lithell et al. 1997; Tark et al. 1989), hand fracture (Nijhuis et al. 2010), peripheral nerve and brachial plexus injury (Novak et al. 2012; Ruijs et al. 2007), upper extremity arterial injury (Klocker et al. 2012), flexor tendon repair (Riaz et al. 1999), corrective surgery for Dupuytren’s disease (McKirdy 2007), carpal tunnel syndrome (Thomsen et al. 2009), freezing cold injury (Carlsson et al. 2014), and hand–arm vibration (HAV) syndrome (Carlsson et al. 2010c; Necking et al. 2002). Injuries aside, cold sensitivity has also been described in relation to diabetes mellitus (Thomsen et al. 2009), and rheumatic diseases (Merkel et al. 2002). In several publications, cold sensitivity was found to be the worst and longest-lasting problem following a hand injury (Carlsson et al. 2010a; Lithell et al. 1997), and was shown to reduce quality of life (Carlsson et al. 2010c; Koman et al. 1998). To our knowledge, cold sensitivity has not previously been investigated in population-based studies.

In occupational health standards, ambient temperatures at or below 10 °C have been defined as cold exposure (International Organization for Standardization 2008). The experience of being cold can also be defined from a subjective standpoint, regardless of ambient temperature (Makinen and Hassi 2002). Cold exposure may occur during both work and leisure-time, and is often associated with aggravating environmental conditions such as wind, rain or snow (Keim et al. 2002). In addition, indoor cold store work, contact with cold objects, and cold water immersion can contribute to the effects of cold (Baldus et al. 2012). These effects are also modified by individual factors such as sex, age, nutritional status, pre-existing diseases, medication, thermal clothing insulation, and activity level (Raatikka et al. 2007). Swedish national statistics from 2015 report that 23% of working men and 14% of working women in Sweden are occupationally exposed to an ambient cold climate for at least one quarter of their working hours (Swedish Work Environment Authority 2016). During leisure-time, 30% of men, and 25% of women, living in Northern Sweden, report a high cold exposure (Stjernbrandt et al. 2017).

The aim of the present study was to identify factors associated with the reporting of cold sensitivity, by comparing cases to controls with regard to anthropometry, previous illnesses and injuries, as well as external exposures such as HAV and ambient cold.

## Methods

### Participants and data collection

In the spring of 2015, a research project called Cold and Health in Northern Sweden (CHINS) was launched, with the purpose of investigating cold-related health effects in Northern Sweden. The project was conducted in the four northernmost counties in Sweden: Norrbotten; Västerbotten; Västernorrland; and Jämtland. The study region holds a population of approximately 880,000 people (Statistics Sweden 2016), and is located between the 62°N and 69°N latitude, with a mixed subarctic and temperate climate.

The first data collection, here titled CHINS1, was initiated on the 5th of February and ended on the 5th of May, 2015. It consisted of a large questionnaire-based study performed on a sample of men and women between ages 18 and 70 years living in the study area. The study sample was randomly selected from the national Swedish population register. The rationale and methodology for the CHINS1 study have previously been described in detail (Stjernbrandt et al. 2017).

From the collected baseline data, cases with cold sensitivity were identified through the use of two questionnaire items:

1. “I am oversensitive to cold” to which the study participant could answer on a fixed numerical scale ranging from 1 (“do not agree”) to 10 (“agree completely”). An answer of 4 or more was considered a positive response.
2. “I experience pain/discomfort when fingers/hands are exposed to cold” to which the study participant could answer on a four-grade scale, in the form of “none”, “insignificant”, “somewhat” or “a lot”. Answering “a lot” was considered a positive response.

A positive response on both questions fulfilled our case definition for cold sensitivity. All cases were invited to participate in a second data collection, here titled CHINS2, which was a questionnaire-based nested case–control study. Controls were randomly selected with a ratio of 2:1 among study subjects from CHINS1 according to the following inclusion criteria:

1. No reported cold sensitivity according to the definition described above.
2. No reported Raynaud’s phenomenon.
3. Matching the case with regard to geographical area, sex, and age (± 2 years).

**Fig. 1:**
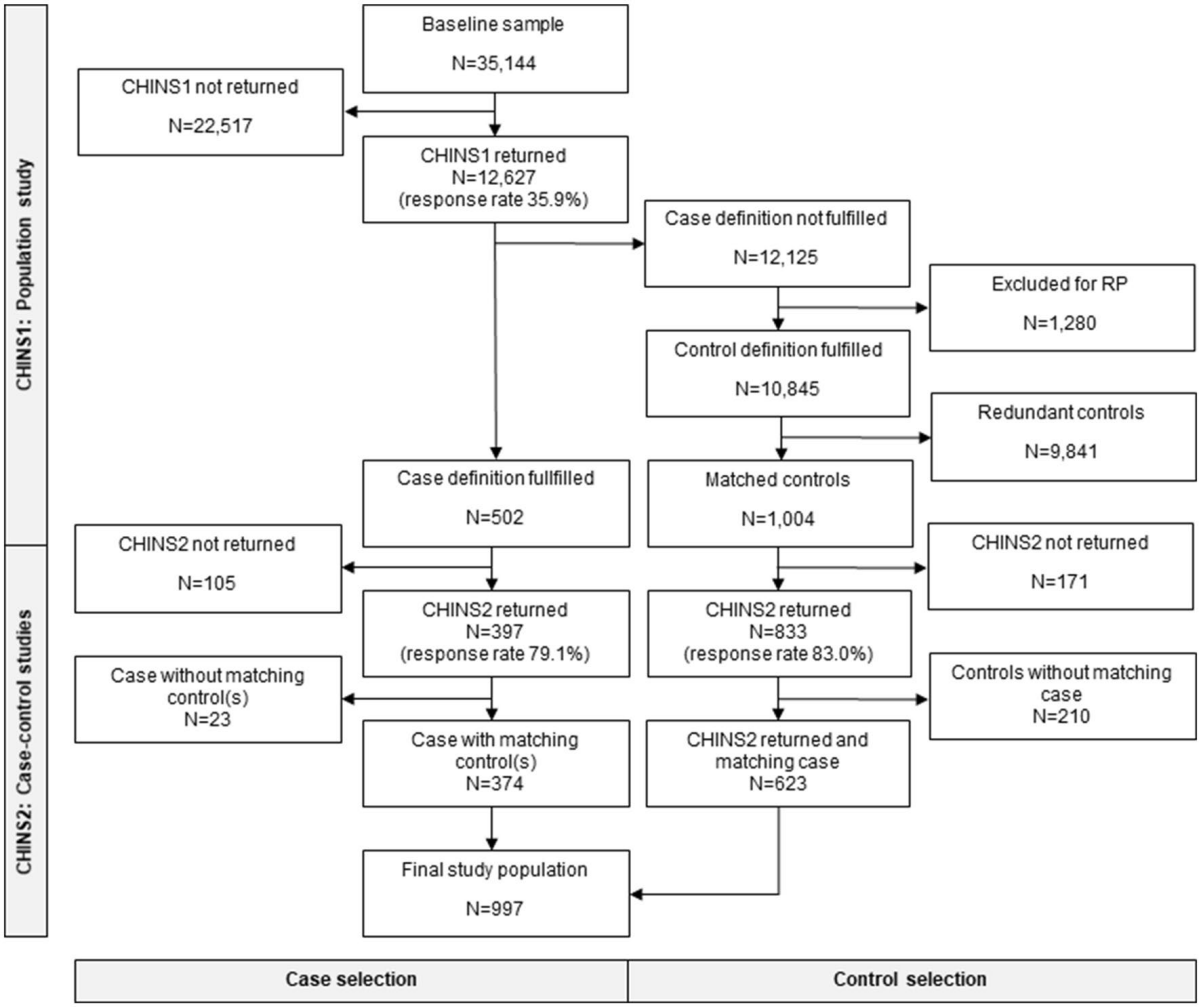
Data collection for the CHINS1 and CHINS2 studies. The number of study subjects in each step of the data collection process is illustrated, and the response rates for each of the questionnaires are shown in parentheses. *CHINS1* the first, population-based, data collection. *CHINS2* the second, case-based, data collection. *CS* cold sensitivity, *RP* Raynaud’s phenomenon

The CHINS2 study was initiated on the 10th of October 2015, and ended on the 10th of March 2016. Cases and controls received the same questionnaire. Details regarding the data collection are presented in Fig. 1. The study protocol was approved by the Regional Ethical Review Board situated at Umeå University (DNR 2015-24-31M and 2014-286-31M).

### Study design

The study questionnaire was designed by a team of physicians, occupational and environmental hygienists, engineers, and ergonomists; and collected data on demographic and anthropometric variables such as place of livelihood, sex, age, height, and weight. Geographical location was determined by postal code and stratified into 44 municipalities that were then grouped together to form three broad categories—coastal, inland, and alpine. The occupations of the study participants were collected in free text, and then coded in accordance with the International Standard Classification of Occupations (International Labour Organization 2012). The use of tobacco, either cigarettes or snuff, was also included.

To quantify the severity of cold sensitivity, we added a 100 mm visual analogue scale (VAS), where the study participants were asked to mark the extent of problems with their hands they experience when exposed to cold climate. We also included the Swedish version of the Cold Intolerance Symptom Severity (CISS) score (Carlsson et al. 2008) in our questionnaire. This inventory scores subjective problems with ambient cold exposure on a scale ranging from 4 to 100, where a value exceeding 50 has been suggested to indicate abnormal cold sensitivity, based on a cohort of randomly selected Swedish healthy volunteers (Carlsson et al. 2010b).

Frostbite affecting the hands was categorized as first degree (white spots), second degree (blisters), or third degree (blood-filled blisters). The occurrence of Raynaud’s phenomenon was investigated through a single item question; “Does one or more of your fingers turn white (as shown on picture) when exposed to moisture or cold?” and was supported by a standardized color chart that has previously been shown to increase the diagnostic specificity (Negro et al. 2008). Other questions asked if the study participants had been diagnosed by a physician for any of the following: hypertension; angina pectoris; myocardial infarction; stroke; diabetes mellitus; joint disease; or migraines. Questions were also posed about the presence of rheumatic disease, upper extremity nerve injury, polyneuropathy, carpal tunnel syndrome, and peripheral vascular disease, and the study participants were asked to specify the condition in detail (in free text).

The use of therapeutic drugs was collected in free text, and coded by one of the study physicians (AS) into two broad categories based on whether the substance has a documented negative effect on either peripheral nerves (Asbury 2006; Chan and Wilder-Smith 2016) or circulation (Bakst et al. 2008; Block and Sequeira 2001). Drugs classified as having a negative effect on peripheral nerves were statins (pravastatin, simvastatin, atorvastatin, rosuvastatin), certain antibiotics (metronidazole, nitrofurantoin, linezolide, isoniazide), certain immunosuppressive drugs (etanercept, infliximab, adalimumab, certolizumab pegol, golimumab, leflunomid), certain antineoplastic agents (cisplatin, taxol, vincristine, oxaliplatin, bortezomib), amiodarone, dapsone, phenytoin, and hydralazine. Drugs considered to have a negative effect on peripheral circulation were beta-adrenergic antagonists (metoprolol, bisoprolol, atenolol, propranolol, pindolol, carvedilol), interferons (alpha and beta), systemic hormone replacement or contraceptive treatment, certain antineoplastic agents (cisplatin, bleomycin, vinblastine, tegafur), certain sympathomimetic drugs (methylamphetamine, dexamphetamine), lithium, clonidine, and ergotamine.

Ambient cold exposure was investigated with several questions, partly rephrased from the Potential Work Exposure Scale (McCabe et al. 1991). For example, study participants were asked if their work required them to manually handle objects with a temperature near or below freezing. They were also asked to grade their occupational and leisure-time cold exposure on a fixed numerical rating scale (NRS) ranging from 1 to 10, respectively. The two scales were subsequently added together to form a cumulative measurement of cold exposure ranging from 2 to 20. For HAV, the study participants were asked to specify if they had recurrent occupational exposure to impact tools (chipping hammers, rotary hammers, rock drills, impact drills, nailers, impact wrenches), rapidly rotating tools (dentist drills, dental technician instruments, foot files), forestry and gardening equipment (chainsaws, brush cutters, lawn mowers, hedge trimmers), vibrating tools (screwdrivers, drilling machines, circular saws, belt sanders), heavily vibrating tools (reciprocating saws, jigsaws, oscillating sanders, soil compactors, concrete vibrators), or vehicles with vibrating controls (graders, tractors, trucks, snowmobiles, all-terrain vehicles).

### Statistical analysis

Descriptive characteristics for cases and controls were presented as means and standard deviation (SD) for continuous variables, and as numbers and percentages for categorical variables. Numerical rating scales for occupational and leisure-time, as well as for cumulative cold exposure, were dichotomized into high or low exposure based on the 50th percentile. The cumulative cold exposure scale was also categorized into quartiles.

Associations between cold sensitivity and each of the candidate factors were assessed separately using univariate conditional logistic regression, and presented as odds ratios of reporting cold sensitivity (Tables 2, 3). *P* values less than 0.05 were considered statistically significant. Thereafter, multiple conditional logistic regression was used to identify the most important associated factors using a manual forward stepwise procedure where, in each step, the associated factor with the lowest *P* value when entered into the model was added (Table 4). *P* values were obtained using the Wald test. Only associated factors with a *P* value less than 0.05 when entered were subsequently added to the model. Sex-specific subgroup analyses were also conducted for both the univariate and multiple models. All statistical analyses were performed using IBM SPSS Statistics for Windows (version 23.0, IBM Corporation, Armonk, NY, USA).

## Results

### Participants (Fig. 1; Table 1)

**Table 1:** Descriptive characteristics for cases and controls as numbers and percentages, presented in total and separated by sex

	All subjects		Men		Women	
	Cases	Controls	Cases	Controls	Cases	Controls
	*N* (%)	*N* (%)	*N* (%)	*N* (%)	*N* (%)	*N* (%)
Responders	374 (37.5)	623 (62.5)	136 (37.3)	229 (62.7)	238 (37.7)	394 (62.3)
Age category (years)						
18–30	38 (10.2)	58 (9.3)	9 (6.6)	13 (5.7)	29 (12.2)	45 (11.4)
30–40	45 (12.0)	62 (10.0)	9 (6.6)	12 (5.2)	36 (15.1)	50 (12.7)
40–50	85 (22.7)	137 (22.0)	21 (15.4)	32 (14.0)	64 (26.9)	105 (26.6)
50–60	105 (28.1)	173 (27.8)	44 (32.4)	70 (30.6)	61 (25.6)	103 (26.1)
60–70	101 (27.0)	193 (31.0)	53 (39.0)	102 (44.5)	48 (20.2)	91 (23.1)
BMI category						
Underweight (BMI < 18.5)	5 (1.4)	5 (0.8)	0 (0)	0 (0)	5 (2.1)	5 (1.3)
Normal weight (18.5 ≤ BMI < 25)	206 (56.0)	261 (43.0)	63 (47.0)	73 (32.6)	143 (61.1)	188 (49.1)
Overweight (25 ≤ BMI < 30)	102 (27.7)	230 (37.9)	48 (35.8)	110 (49.1)	54 (23.1)	120 (31.3)
Obese (BMI ≥ 30)	55 (14.9)	111 (18.3)	23 (17.2)	41 (18.3)	32 (13.7)	70 (18.3)
Tobacco use						
Daily cigarette smoking	33 (8.9)	49 (7.9)	9 (6.7)	19 (8.3)	24 (10.1)	30 (7.7)
Daily snuff use	57 (15.4)	66 (10.7)	37 (27.4)	46 (20.3)	20 (8.5)	20 (5.2)
Any daily tobacco use	89 (23.8)	107 (17.2)	45 (33.1)	61 (26.6)	44 (18.5)	46 (11.7)
Area of livelihood						
Alpine	87 (23.3)	146 (23.4)	33 (24.3)	60 (26.2)	54 (22.7)	86 (21.8)
Inland	110 (29.4)	176 (28.3)	46 (33.8)	72 (31.4)	64 (26.9)	104 (26.4)
Coastal	177 (47.3)	301 (48.3)	57 (41.9)	97 (42.4)	120 (50.4)	204 (51.8)
Occupation						
Armed forces occupations	2 (0.6)	3 (0.5)	1 (0.8)	2 (0.9)	1 (0.4)	1 (0.3)
Managers	9 (2.5)	22 (3.6)	6 (4.5)	7 (3.1)	3 (1.3)	15 (3.9)
Professionals	73 (20.2)	115 (19.0)	17 (12.9)	24 (10.7)	56 (24.5)	91 (23.9)
Technicians and associate professionals	28 (7.8)	70 (11.6)	14 (10.6)	34 (15.2)	14 (6.1)	36 (9.4)
Clerical support workers	40 (11.1)	47 (7.8)	10 (7.6)	12 (5.4)	30 (13.1)	35 (9.2)
Service and sales workers	57 (15.8)	107 (17.7)	11 (8.3)	30 (13.4)	46 (20.1)	77 (20.2)
Skilled agricultural, forestry, and fishery workers	6 (1.7)	9 (1.5)	2 (1.5)	3 (1.3)	4 (1.7)	6 (1.6)
Crafts and related trades workers	17 (4.7)	26 (4.3)	14 (10.6)	19 (8.5)	3 (1.3)	7 (1.8)
Plant and machine operators and assemblers	27 (7.5)	27 (4.5)	22 (16.7)	22 (9.8)	5 (2.2)	5 (1.3)
Elementary occupations	6 (1.7)	13 (2.1)	3 (2.3)	5 (2.2)	3 (1.3)	8 (2.1)
Self-employed	8 (2.2)	13 (2.1)	2 (1.5)	8 (3.6)	6 (2.6)	9 (2.4)
Students	11 (3.0)	16 (2.6)	1 (0.8)	0 (0)	10 (4.4)	16 (4.2)
Unemployed	4 (1.1)	9 (1.5)	2 (1.5)	4 (1.8)	2 (0.9)	5 (1.3)
Parental leave	3 (0.8)	4 (0.7)	0 (0)	0 (0)	3 (1.3)	4 (1.0)
Sick leave	12 (3.3)	12 (2.0)	1 (0.8)	3 (1.3)	11 (4.8)	9 (2.4)
Retired	58 (16.1)	112 (18.5)	26 (19.7)	51 (22.8)	32 (14.0)	61 (16.0)

*BMI* body mass index

The case definition for cold sensitivity was fulfilled by 502 participants (4.0%) in the CHINS1 dataset, and they were all invited to participate in the nested case–control study. Also from the CHINS1 dataset, 10,845 eligible controls were identified, of which 1004 randomly selected matched controls were invited to participate. Of the 502 cases, 397 (79.1%) returned the CHINS2 questionnaire, and of the 1004 controls, 833 (83.0%) responded. In total, 1230 out of 1506 questionnaires were returned, giving an overall response rate of 81.7%. However, 23 responding cases lacked at least one matching responding control, and were subsequently excluded from analyses. A further 210 controls lacking responding cases were also excluded. The final study population consisted of 997 individuals, of which 374 were cases and 623 matching controls. All cases had at least one matching control. The data collection is described in detail in Fig. 1.

### Study population characteristics (Table 1)

The final study population had a predominance of women (63.6% of cases and 63.2% of controls). Cases were comparable to controls with regard to age (mean 50.5 and 51.8 years, respectively), as well as geographical and occupational distribution pattern (Table 1). Body mass index (BMI) was also comparable for cases and controls (mean 25.4 and 26.3 kg/m^2^). According to the Swedish version of CISS, 146 (46.6%) cases and 31 (6.6%) controls exceeded the cut-off value for abnormal cold sensitivity. Cases graded their cold sensitivity higher on VAS (mean 77.8, SD 19.2) than controls (mean 28.9, SD 27.4). Lifetime-occurrence of frostbite affecting the hands was reported by 114 cases (30.5%), and 34 controls (5.5%) of controls (*N* = 34), of which most were first degree injuries (95.5% and 97.0%, respectively). Raynaud’s phenomenon was reported by 61.5% (*N* = 228) of cases, and null among controls (exclusion criteria).

### Univariate conditional logistic regression analyses (Tables 2, 3)

**Table 2:** Univariate conditional logistic regression of factors suggested to be associated with cold sensitivity, including BMI, tobacco use, therapeutic drug use, diseases, and injuries

Factor	Exposure level	All subjects			Men			Women		
		Cases	Controls	OR (95% CI)	Cases	Controls	OR (95% CI)	Cases	Controls	OR (95% CI)
		*N* (%)	*N* (%)		*N* (%)	*N* (%)		*N* (%)	*N* (%)	
BMI category	BMI < 18.5	5 (1.4)	5 (0.8)	1.6 (0.4–6.7)	0 (0.0)	0 (0.0)	–	5 (2.1)	5 (1.3)	1.6 (0.4–6.7)
	18.5 ≤ BMI < 25	206 (56.0)	261 (43.0)	Reference	63 (47.0)	73 (32.6)	Reference	143 (61.1)	188 (49.1)	Reference
	25 ≤ BMI < 30	102 (27.7)	230 (37.9)	0.5 (0.4–0.7)*	48 (35.8)	110 (49.1)	0.5 (0.3–0.8)*	54 (23.1)	120 (31.3)	0.6 (0.4–0.8)*
	BMI ≥ 30	55 (14.9)	111 (18.3)	0.6 (0.4–9)*	23 (17.2)	41 (18.3)	0.6 (0.3–1.1)	32 (13.7)	70 (18.3)	0.6 (0.4–0.96)*
Any daily tobacco use	Yes	89 (23.8)	107 (17.2)	1.5 (1.1–2.0)*	45 (33.1)	61 (26.6)	1.3 (0.9–2.1)	44 (18.5)	46 (11.7)	1.6 (1.04–2.6)*
	No	285 (76.2)	516 (82.8)	Reference	91 (66.9)	168 (73.4)	Reference	194 (81.5)	348 (88.3)	Reference
Therapeutic drug use										
Affecting peripheral nerves^a^	Yes	33 (11.1)	54 (12.3)	1.0 (0.6–1.7)	19 (18.3)	31 (20.3)	0.7 (0.4–1.6)	14 (7.3)	23 (8.1)	1.3 (0.6-3.0)
	No	263 (88.9)	384 (87.7)	Reference	85 (81.7)	122 (79.7)	Reference	178 (92.7)	262 (91.9)	Reference
Affecting peripheral circulation^b^	Yes	55 (19.1)	81 (18.9)	1.3 (0.8–2.1)	22 (22.0)	24 (16.4)	1.7 (0.7–3.7)	33 (17.6)	57 (20.1)	1.1 (0.6-2.0)
	No	233 (80.9)	348 (81.1)	Reference	78 (78.0)	122 (83.6)	Reference	155 (82.4)	226 (79.9)	Reference
Diseases and injuries										
Vascular disease^c^	Yes	109 (29.1)	151 (25.5)	1.8 (1.3–2.6)*	50 (41.0)	70 (32.4)	2.2 (1.2–3.9)*	59 (26.6)	81 (21.6)	1.6 (1.04–2.5)*
	No	235 (68.3)	440 (74.5)	Reference	72 (59.0)	146 (67.6)	Reference	163 (73.4)	294 (78.4)	Reference
Diabetes mellitus	Yes	13 (3.5)	28 (4.6)	0.9 (0.5–1.8)	6 (4.4)	12 (5.4)	0.9 (0.3–2.4)	7 (3.0)	16 (4.1)	0.9 (0.4–2.3)
	No	356 (96.5)	581 (95.4)	Reference	129 (95.6)	211 (94.6)	Reference	227 (97.0)	370 (95.9)	Reference
Migraines	Yes	59 (16.1)	50 (8.2)	2.2 (1.4–3.3)*	12 (9.0)	10 (4.4)	2.0 (0.9–4.7)	47 (20.2)	40 (10.5)	2.2 (1.4–3.6)*
	No	307 (83.9)	558 (91.8)	Reference	121 (91.0)	216 (95.6)	Reference	186 (79.8)	342 (89.5)	Reference
Rheumatic disease^d^	Yes	66 (18.1)	45 (7.4)	3.2 (2.1-5.0)*	15 (11.4)	16 (7.1)	2.0 (0.9–4.3)	51 (21.9)	29 (7.6)	3.9 (2.3–6.8)*
	No	299 (81.9)	562 (92.6)	Reference	117 (88.6)	208 (92.9)	Reference	182 (78.1)	354 (92.4)	Reference
Carpal tunnel syndrome	Yes	39 (10.9)	50 (8.2)	1.5 (0.9–2.3)	13 (10.0)	19 (8.5)	1.3 (0.6-3.0)	26 (11.4)	31 (8.1)	1.6 (0.9–2.7)
	No	320 (89.1)	559 (91.8)	Reference	117 (90.0)	205 (91.5)	Reference	203 (88.6)	354 (91.9)	Reference
Polyneuropathy	Yes	13 (3.6)	4 (0.7)	7.4 (2.1–26.4)*	7 (5.3)	2 (0.9)	10.2 (1.2–85.4)*	6 (2.6)	2 (0.5)	6.0 (1.2–29.7)*
	No	350 (96.4)	602 (99.3)	Reference	125 (94.7)	222 (99.1)	Reference	225 (97.4)	380 (99.5)	Reference
Upper extremity nerve injury	Yes	119 (32.6)	112 (18.3)	2.3 (1.7–3.1)*	50 (37.6)	47 (20.6)	2.3 (1.4–3.7)*	69 (29.7)	65 (16.9)	2.3 (1.5–3.5)*
	No	246 (67.4)	500 (81.7)	Reference	83 (62.4)	181 (79.4)	Reference	163 (70.3)	319 (83.1)	Reference
Frostbite hands	Yes	114 (30.6)	34 (5.5)	**10.2 (6.0–17.2**)*	53 (39.0)	13 (5.7)	**18.9 (6.8–52.6**)*	61 (25.8)	21 (5.4)	7.2 (3.9–13.6)*
	No	258 (69.4)	583 (94.5)	Reference	83 (61.0)	215 (94.3)	Reference	175 (74.2)	368 (94.6)	Reference

Data presented in total and separated by sex

*BMI* body mass index

*Bold values indicate odds ratios with significant 95% confidence intervals

^a^Statins, antibiotics, immunosuppressive drugs, antineoplastic agents, amiodarone, dapsone, phenytoin and/or hydralazine

^b^Beta-adrenergic antagonists, interferons, systemic hormone replacement or contraceptive treatment, antineoplastic agents, sympathomimetics drugs, lithium, clonidine, and/or ergotamine

^c^Hypertension, angina pectoris, myocardial infarction, stroke, and/or peripheral vascular disease

^d^Systemic sclerosis, CREST syndrome, rheumatoid arthritis, juvenile rheumatoid arthritis, reactive arthritis, unspecified arthritis, systemic lupus erythematosus, psoriatic arthritis, ankylosing spondylitis, Sjogren’s syndrome, Ehlers–Danlos syndrome, fibromyalgia, gout, polymyositis, dermatomyositis, Dercum’s disease, and/or mixed connective tissue disease

**Table 3:** Univariate conditional logistic regression of external exposure factors suggested to be associated with cold sensitivity, including different cold and hand–arm vibration exposure measures

Factor	Exposure level	All subjects			Men			Women		
		Cases	Controls	OR (95% CI)	Cases	Controls	OR (95% CI)	Cases	Controls	OR (95% CI)
		*N* (%)	*N* (%)		*N* (%)	*N* (%)		*N* (%)	*N* (%)	
Cold exposure measures										
Occupational cold exposure^a^ (NRS 1–10)	High (NRS > 1)	182 (50.4)	242 (40.8)	1.4 (1.1–1.9)*	88 (67.7)	132 (60.8)	1.3 (0.8–2.1)	94 (40.7)	110 (29.3)	1.5 (1.1–2.2)*
	Low (NRS 1)	179 (49.6)	351 (59.2)	Reference	42 (32.3)	85 (39.2)	Reference	137 (59.3)	266 (70.7)	Reference
Leisure-time cold exposure^b^ (NRS 1–10)	High (NRS > 5)	194 (52.9)	301 (49.3)	1.1 (0.8–1.4)	69 (51.1)	118 (52.0)	1.0 (0.6–1.4)	125 (53.9)	183 (47.8)	1.2 (0.9–1.7)
	Low (NRS ≤ 5)	173 (47.1)	309 (50.7)	Reference	66 (48.9)	109 (48.0)	Reference	107 (46.1)	200 (52.2)	Reference
Cumulative cold exposure^c^ (NRS 2–20)	High (NRS > 8)	198 (55.3)	253 (43.0)	1.6 (1.2–2.1)*	81 (62.3)	122 (56.5)	1.2 (0.7–1.9)	117 (51.3)	131 (35.2)	1.8 (1.3–2.6)*
	Low (NRS ≤ 8)	160 (44.7)	335 (57.0)	Reference	49 (37.7)	94 (43.5)	Reference	111 (48.7)	241 (64.8)	Reference
Cumulative cold exposure^d^ (NRS 2–20)	1st quartile	69 (19.3)	141 (24.0)	Reference	22 (16.9)	34 (15.7)	Reference	47 (20.6)	107 (28.8)	Reference
	2nd quartile	91 (25.4)	194 (33.0)	1.0 (0.7–1.5)	27 (20.8)	60 (27.8)	0.8 (0.4–1.6)	64 (28.1)	134 (36.0)	1.1 (0.7–1.8)
	3rd quartile	110 (30.7)	147 (25.0)	1.5 (1.04–2.3)*	36 (27.7)	62 (28.7)	0.97 (0.5–1.9)	74 (32.5)	85 (22.8)	**1.9 (1.2–3.1)**
	4th quartile	88 (24.6)	106 (18.0)	1.6 (1.1–2.5)*	45 (34.6)	60 (27.8)	1.1 (0.6–2.2)	43 (18.9)	46 (12.4)	1.9 (1.1–3.4)*
Handling cold objects during work	Yes	123 (33.5)	108 (17.6)	2.6 (1.9–3.6)*	77 (57.0)	73 (32.0)	2.8 (1.8–4.5)*	46 (19.8)	35 (9.1)	2.4 (1.5–3.9)*
	No	244 (66.5)	506 (82.4)	Reference	58 (43.0)	155 (68.0)	Reference	186 (80.2)	351 (90.9)	Reference
Extreme cold, wind or cooling moist during work	Yes	133 (36.3)	146 (23.8)	2.3 (1.7–3.3)*	85 (63.9)	103 (45.2)	2.4 (1.5–3.9)*	48 (20.6)	43 (11.1)	2.2 (1.4–3.6)*
	No	233 (63.7)	468 (76.2)	Reference	48 (36.1)	125 (54.8)	Reference	185 (79.4)	343 (88.9)	Reference
Occupational HAV exposure measures										
Impact tools	Yes	56 (15.2)	47 (7.7)	2.4 (1.5–3.9)*	50 (37.3)	40 (17.5)	2.8 (1.7–4.6)*	6 (2.6)	7 (1.8)	1.3 (0.4-4.0)
	No	312 (84.8)	567 (92.3)	Reference	84 (62.7)	188 (82.5)	Reference	228 (97.4)	379 (98.2)	Reference
Rapidly rotating tools	Yes	14 (3.7)	11 (1.8)	1.9 (0.9–4.2)	10 (7.6)	7 (3.1)	2.3 (0.9-6.0)	4 (1.7)	4 (1.0)	1.3 (0.3–5.3)
	No	352 (96.2)	602 (98.2)	Reference	122 (92.4)	220 (96.9)	Reference	230 (98.3)	382 (99.0)	Reference
Forestry/gardening tools	Yes	53 (14.5)	75 (12.3)	1.4 (0.9–2.2)	47 (35.3)	68 (29.8)	1.4 (0.9–2.4)	6 (2.6)	7 (1.8)	1.3 (0.4-4.0)
	No	313 (85.5)	537 (87.7)	Reference	86 (64.7)	160 (70.2)	Reference	227 (97.4)	377 (98.2)	Reference
Vibrating tools	Yes	62 (16.8)	65 (10.6)	1.9 (1.2–2.9)*	54 (40.6)	58 (25.6)	2.0 (1.2–3.2)*	8 (3.4)	7 (1.8)	1.5 (0.5–4.2)
	No	306 (83.2)	548 (89.4)	Reference	79 (59.4)	169 (74.4)	Reference	227 (96.6)	379 (98.2)	Reference
Heavily vibrating tools	Yes	54 (14.7)	52 (8.5)	2.4 (1.5–3.9)*	51 (38.3)	49 (21.7)	2.5 (1.5–4.1)*	3 (1.3)	3 (0.8)	1.6 (0.3–8.1)
	No	313 (85.3)	560 (91.5)	Reference	82 (61.7)	177 (78.3)	Reference	177 (78.3)	383 (99.2)	Reference
Vehicles with vibrating controls	Yes	50 (13.6)	66 (10.8)	1.5 (0.95–2.3)	42 (31.6)	58 (25.4)	1.5 (0.9–2.4)	8 (3.4)	8 (2.1)	1.6 (0.6–4.2)
	No	317 (86.4)	547 (89.2)	Reference	91 (68.4)	170 (74.6)	Reference	226 (96.6)	377 (97.9)	Reference
Any HAV exposure^e^	Yes	111 (30.3)	131 (21.4)	2.1 (1.4–3.0)*	89 (66.4)	107 (46.9)	2.6 (1.6–4.3)*	22 (9.5)	24 (6.2)	1.4 (0.8–2.6)
	No	255 (69.7)	482 (78.6)	Reference	45 (33.6)	121 (53.1)	Reference	210 (90.5)	361 (93.8)	Reference

Data presented in total and separated by sex

*NRS* numerical rating scale, *HAV* hand–arm vibration

*Bold values indicate odds ratios with significant 95% confidence intervals

^a^Self-estimated occupational cold exposure, reported on a ten-point numerical rating scales (NRS), where a value above the 50th percentile (NRS > 1) was denoted high, while a value below (NRS 1) was denoted low

^b^Self-estimated leisure-time cold exposure, reported on a ten-point numerical rating scales (NRS), where a value above the 50th percentile (NRS > 5) was denoted high, while a value below (NRS ≤ 5) was denoted low

^c^Self-estimated occupational and leisure-time cold exposure, reported on two separate ten-point numerical rating scales (NRS), were added together to form a cumulative measurement of cold exposure ranging from 2 to 20, and a value above the 50th percentile (NRS > 8) was denoted high, while a value below (NRS ≤ 8) was denoted low

^d^First quartile corresponds to NRS 2–5, second quartile NRS 6–8, third quartile NRS 9–11, and forth quartile NRS 12–20

^e^Any occupational use of impact tools, rapidly rotating tools, forestry and gardening tools, vibrating tools, heavily vibrating tools, and/or vehicles with vibrating controls

Regarding individual factors, being overweight (25 ≤ BMI < 30) was associated with a lower reported frequency of cold sensitivity (OR 0.5, 95% CI 0.4–0.7) compared to being normal weight (18.5 ≤ BMI < 25 kg/m^2^). Any daily use of tobacco was associated with cold sensitivity (OR 1.5, 95% CI 1.1–2.0), but this was not statistically significant among men when analyzed separately. The use of therapeutic drugs with documented harmful effects on peripheral nerves or circulation did not show any association with cold sensitivity. The cold sensitivity cases reported a higher frequency of vascular disease (OR 1.8, 95% CI 1.3–2.6), polyneuropathy (OR 7.4, 95% CI 2.1–26.4), upper extremity nerve injury (OR 2.3, 95% CI 1.7–3.1), and frostbite affecting the hands (OR 10.2, 95% CI 6.0–17.2). This relationship was also seen when analyzing men and women separately. Cases also reported a higher prevalence of migraines (OR 2.2, 95% CI 1.4–3.3), and rheumatic disease (OR 3.2, 95% 2.1–5.0), but this was not statistically significant among men when sex-specific subgroup analyses were performed.

For ambient factors, handling cold objects at work was associated with cold sensitivity (OR 2.6, 95% CI 1.9–3.6), as well as exposure to extreme cold, wind, or cooling moisture during work (OR 2.3, 95% CI 1.7–3.3). These ambient factors were also associated among both men and women, analyzed separately. A high occupational cold exposure (above the 50th percentile, translating to NRS > 1 on a scale ranging from 1 to 10) was associated with reporting cold sensitivity (OR 1.4, 95% CI 1.1–1.9), as was a high cumulative cold exposure (above the 50th percentile, translating to NRS > 8 on a scale ranging from 2 to 20) with an OR of 1.6 (95% CI 1.2–2.1). However, these findings were not statistically significant for men. When the cumulative cold exposure measure was divided into quartiles, a dose–response trend was discernible but not statistically significant in every subgroup. The use of impact tools (OR 2.4, 95% CI 1.5–3.9), vibrating tools (OR 1.9, 95% CI 1.2–2.9), and heavily vibrating tools (OR 2.4, 95% CI 1.5–3.9) all showed associations with cold sensitivity, but only among men.

### Multiple conditional logistic regression analyses (Table 4)

**Table 4:** Manual forward stepwise multiple conditional logistic regression of factors associated with cold sensitivity in the univariate analyses

Factor	Exposure level	Cases		Controls		OR	(95% CI)
		*N*	%	*N*	%		
All subjects							
Frostbite hands	Yes	97	30	32	6	10.3	(5.5–19.3)*
	No	225	70	527	94	Reference	–
Rheumatic disease^a^	Yes	58	18	36	6	**3.1**	(1.7–5.7)*
	No	264	82	523	94	Reference	–
BMI category (kg/m^2^)	BMI < 18.5	5	2	5	1	1.1	(0.1–9.9)
	18.5 ≤ BMI < 25	185	57	243	43	Reference	–
	BMI ≥ 25	132	41	311	56	**0.4**	(0.3–0.6)*
Upper extremity nerve injury	Yes	98	30	99	18	**2.0**	(1.3-3.0)*
	No	224	70	460	82	Reference	–
Migraines	Yes	51	16	42	8	**2.4**	(1.3–4.3)*
	No	271	84	517	92	Reference	–
Vascular disease^b^	Yes	101	31	142	25	**1.9**	(1.2–2.9)*
	No	221	69	417	75	Reference	–
Men							
Frostbite hands	Yes	51	39	13	6	**17.9**	(6.1–52.1)*
	No	79	61	209	94	Reference	–
Any HAV exposure^c^	Yes	87	67	103	46	**2.2**	(1.2–4.2)*
	No	43	33	119	54	Reference	–
BMI category (kg/m^2^)	BMI < 18.5	0	0	0	0	–	–
	18.5 ≤ BMI < 25	62	48	73	33	Reference	–
	BMI ≥ 25	68	52	149	67	**0.4**	(0.2–0.8)*
Upper extremity nerve injury	Yes	49	38	46	21	**2.4**	(1.2–4.6)*
	No	81	62	176	79	Reference	–
Women							
Frostbite hands	Yes	53	25	19	6	**7.6**	(3.5–16.6)*
	No	159	75	326	94	Reference	–
Rheumatic disease^a^	Yes	45	21	23	7	**4.2**	(1.9-9.0)*
	No	167	79	322	93	Reference	–
BMI category (kg/m^2^)	BMI < 18.5	5	2	5	1	1.5	(0.2–13.4)
	18.5 ≤ BMI < 25	130	61	168	49	Reference	–
	BMI ≥ 25	77	36	172	50	**0.5**	(0.3–0.8)*
Upper extremity nerve injury	Yes	57	27	55	16	1.5	(0.9–2.7)
	No	155	73	290	84	Reference	–
Migraines	Yes	42	20	34	16	**2.3**	(1.2–4.5)*
	No	170	80	311	84	Reference	–
Cumulative cold exposure^d^	High (NRS > 8)	106	50	119	34	**1.6**	(1.04–2.4)*
	Low (NRS ≤ 8)	106	50	226	66	Reference	–

Data presented in total and separated by sex

*BMI* body mass index, *HAV* hand–arm vibration

*Bold values indicate odds ratios with significant 95% confidence intervals

^a^Systemic sclerosis, CREST syndrome, rheumatoid arthritis, juvenile rheumatoid arthritis, reactive arthritis, unspecified arthritis, systemic lupus erythematosus, psoriatic arthritis, ankylosing spondylitis, Sjogren’s syndrome, Ehlers–Danlos syndrome, fibromyalgia, gout, polymyositis, dermatomyositis, Dercum’s disease, and/or mixed connective tissue disease

^b^Hypertension, angina pectoris, myocardial infarction, stroke, and/or peripheral vascular disease

^c^Any occupational use of impact tools, rapidly rating tools, forestry and gardening tools, vibrating tools, heavily vibrating tools, and/or vehicles with vibrating controls

^d^Self-estimated occupational and leisure-time cold exposure, reported on two separate ten-point numerical rating scales (NRS), were added together to form a cumulative measurement of cold exposure ranging from 2 to 20, and a value above the 50th percentile (NRS > 8) was denoted high, while a value below (NRS ≤ 8) was denoted low

In the multiple model for all cases, cold sensitivity was associated with frostbite affecting the hands (OR 10.3, 95% CI 5.5–19.3), rheumatic disease (OR 3.1, 95% CI 1.7–5.7), upper extremity nerve injury (OR 2.0, 95% CI 1.3–3.0), migraines (OR 2.4, 95% CI 1.3–4.3), and vascular disease (OR 1.9, 95% CI 1.2–2.9). Subjects with BMI ≥ 25 kg/m^2^ were less likely to report cold sensitivity than those of normal weight (OR 0.4, 95% CI 0.3–0.6).

Among men, cold sensitivity was associated with frostbite affecting the hands (OR 17.9, 95% CI 6.1–52.1), any HAV exposure (OR 2.2, 95% CI 1.2–4.2), and upper extremity nerve injury (OR 2.4, 95% CI 1.2–4.6). Men with BMI ≥ 25 kg/m^2^ were also less likely to report cold sensitivity than normal-weight subjects (OR 0.4, 95% CI 0.2–0.8).

Women reporting cold sensitivity showed associations with frostbite affecting the hands (OR 7.6, 95% CI 3.5–16.6), rheumatic disease (OR 4.2, 95% CI 1.9–9.0), migraines (OR 2.3, 95% CI 1.2–4.5), and a high cumulative cold exposure (above the 50th percentile, translating to NRS > 8 on a scale ranging from 2 to 20) (OR 1.6, 95% CI 1.04–2.4). In conformity with the other results, a BMI ≥ 25 showed an inverse relationship (OR 0.5, 95% CI 0.3–0.8) to reporting cold sensitivity.

## Discussion

### Key results

The present study shows that cold sensitivity, on a population level, is associated with several individual and external exposure factors and that the associations are somewhat dependent on sex. In the multiple conditional logistic regression model, previous occurrence of frostbite affecting the hands had the strongest association with reporting increased cold sensitivity for both men and women. Being overweight seemed to be a protection against reporting cold sensitivity for both sexes. For men, HAV exposure and upper extremity nerve injury were positively associated with cold sensitivity, while women showed associations with rheumatic disease, migraines, and cumulative cold exposure. Vascular disease was statistically associated with cold sensitivity only when men and women were analyzed together.

### Interpretation and comparison

The case definition in this study was fulfilled predominantly by non-smoking middle-aged women. Some previous studies have reported associations between cold sensitivity and age (Schlenker et al. 1980), gender (Ruijs et al. 2007), and tobacco use (Irwin et al. 1997), while others have not found such relations (Collins et al. 1996; Craigen et al. 1999; Nancarrow et al. 1996). Our nested case–control study design with matching did not allow analyses on age and gender to be made, but univariate analyses supported the notion that tobacco use can aggravate cold sensitivity, possibly through a vasoconstrictive mechanism mediated by nicotine. The use of drugs with negative effects on peripheral nerve function and circulation did not differ significantly between cases and controls. Thus, the adverse effects of medication are probably not the primary explanation for cold sensitivity. An alternative view may be that the results are skewed by discontinuation of such drugs in cold sensitivity cases, e.g., beta-adrenergic antagonists being exchanged for other antihypertensive drugs in patients who report cold hands. A high BMI was inversely associated with cold sensitivity, which suggests that it acts as a protective factor, possibly through a passive insulating mechanism.

Frostbite was very common in our case population, and increased cold sensitivity is a recognized sequela in individuals with previous cold injury (Thomas and Oakley 2001). Local cold injuries are traditionally categorized into freezing cold injuries (such as frostbite), occurring at temperatures below 0 °C, and non-freezing cold injuries (such as chilblains) which occur at temperatures above 0 °C and often in conjunction with moisture and local pressure (Imray and Oakley 2005). In this study, ambient cold exposure for women, and previous frostbite occurrence in both men and women were generally more pronounced among cases than controls, which adds to the increasing body of data supporting cold exposure as being a cause of cold sensitivity. However, in an earlier study we demonstrated that high-cumulative ambient cold exposure in the general population is positively related to symptoms of cold sensitivity, even in the absence of overt cold injury (Stjernbrandt et al. 2017). A recently published study on heavily cold-exposed Swedish military conscripts showed a significant increase in symptoms of cold sensitivity after winter training, present also in subjects where no cold injury had been reported (Carlsson et al. 2016). Earlier reports from the Falklands War revealed marked cold sensitivity in British servicemen with mild or even subclinical cases of cold injury (Thomas and Oakley 2001). Hence, at the present time there is not enough data to establish a safe lower limit for ambient cold exposure, and the traditional classification of cold injuries into freezing and non-freezing does not seem to aid in the recognition of cold sensitivity development.

In our univariate analyses, HAV exposure of any kind showed an association with cold sensitivity among men, but not in women. This sex difference is suspected to be due to a small sample of exposed women, causing issues with statistical power. When looking at subgroups of vibrating equipment, the tools commonly recognized to have the most harmful effects (impact tools and heavily vibrating tools) were the ones that showed significant associations with cold sensitivity. Thus, our findings support previous studies reporting a relationship between HAV exposure and cold sensitivity (Carlsson et al. 2010c; Necking et al. 2002).

There were several diseases and injuries that were more prevalent among the cold sensitivity cases than the controls in the present study. Upper extremity nerve injuries were particularly common, and this is in line with previous research (Engkvist et al. 1985; Nylander et al. 1987). Some authors have argued that nerve injury should be considered the main determinant of cold sensitivity (Ruijs et al. 2007), while others have argued that in traumatic hand injury, both vascular, neural, humoral, and bony components can been associated with cold sensitivity (Carlsson and Dahlin 2014). The presence of vascular disease showed a relationship with cold sensitivity in our study, which would support the theory of a vascular mechanism. This finding was seen in all univariate analyses, but only when men and women were grouped together in the multiple analyses. Furthermore, female cases had a significantly higher frequency of rheumatic disease than controls. Among patients with rheumatic disease, both cold sensitivity (Merkel et al. 2002) and Raynaud’s phenomenon (Garner et al. 2015) have previously been reported as a common complaint. In the present study, there was also a very high prevalence of Raynaud’s phenomenon among cases. Thus, cold sensitivity and Raynaud’s phenomenon seem to be heavily overlapping conditions that may share some pathophysiological mechanisms, which warrants further research. One novel finding in our study was the association between migraines and cold sensitivity (OR 2.4, 95% CI 1.3–4.3 in the multiple analyses for all subjects). Some studies have reported a dysfunctional vasoregulatory response to cold exposure in cold sensitive subjects, with increased vasoconstriction (Hope et al. 2014) or abnormal baroreceptor response (Marchant et al. 1994). One may thus hypothesize that cold sensitivity is not primarily related to atherosclerotic vascular disease, but rather to a dysfunctional vasoregulatory system, in which neural function also may play an important role.

Abnormal cold sensitivity according to CISS (score > 50) was seen in 46.6% of cases and 6.6% of controls in the present study, all selected from the general population. This supports our case selection criteria being relevant. In another Swedish study using the same inventory and cut-off value, abnormal cold sensitivity was seen in 75.0% of patients with HAV injury, 51.0% of patients with previous amputation injury, 37.1% of patients with nerve injury, and 4.9% of healthy controls (Carlsson et al. 2010c), closely resembling our results. A simpler but less validated approach to grading the condition would be the use of the VAS, which is easy to report and gives an intuitive result. In the present study, VAS showed a clear distinction between cases and controls, supporting its usefulness.

The experience of cold sensitivity is influenced by psychological factors (Carlsson et al. 2010a), and recent laboratory studies have shown a more pronounced pain response to a cold pressor test among individuals with high anxiety sensitivity (Dodo and Hashimoto 2017). Additionally, perception thresholds to cold and pain are often assessed by psychophysical methods, where the responses are modulated by psychological factors (Carlsson et al. 2016). The present study did not include psychological variables, and this topic remains an important issue for further research.

### Limits

There are several limitations to our study. The nested case–control study design does not allow causal relations to be established. The response rate to the initial questionnaire (CHINS1) was low (35.9%), and as only 4.0% of that group subsequently fulfilled our case definition for the second questionnaire (CHINS2), the cases are highly selected. There is no universally established definition of cold sensitivity, and the condition seems to overlap with Raynaud’s phenomenon, which makes the diagnosis difficult to establish. Our cases reported a high occurrence of Raynaud’s phenomenon, but this was excluded for among controls, which could possibly have introduced a systematic bias into our results. However, since the expected frequency of Raynaud’s phenomenon among healthy controls is low, we believe this possible effect to be rather weak. The cold exposure estimates in our study are defined from a subjective standpoint. These conditions limit the generalizability of the results and increase the uncertainty in risk estimates. There are several possible reporting biases; firstly, there is a possibility that symptomatic subjects might be more prone to respond to questionnaires of this kind, and this might lead to an overestimation of both exposure and symptoms; secondly, there is a risk that a selection effect diminishes the cold exposure estimates in cold sensitive cases, since one would expect that such individuals leave cold-exposed occupations, and are deterred from leisure-time cold exposure as well, as was the case in previous studies (Carlsson and Dahlin 2014). The number of retired respondents was high, which might weaken any possible associations with occupational factors. The study region comprises a large area with a mean monthly temperature during the study period that spanned from about − 9 to 5 °C during the initial data collection, meaning that there is reason to suspect a variance in ambient cold exposure that has not been adjusted for in the analyses. Thus, the results in our study can be used to generate hypotheses regarding the mechanisms behind cold sensitivity, but must be cautiously interpreted with regard to limitations in the study design.

### Strengths

However, to our knowledge this was the first population-based study on cold sensitivity, and it included almost a thousand participants. The anthropometric data, tobacco use, and disease spectrum in our cohort roughly corresponded with other recent Swedish investigations (Eriksson et al. 2011), which indicates that our study has included a representative sample of the population. Our previously published non-responder analysis revealed no major differences between responders and non-responders regarding geographical region, which was the expected main determinant of cold exposure variables (Stjernbrandt et al. 2017). Thus the possible bias introduced by a low response rate in the first questionnaire (CHINS1) is not believed to have affected the exposure data in the present study to any larger extent. The cold sensitivity questionnaire (CHINS2) was sent out during the coldest period of the year, which should lessen the risk of recall bias regarding ambient cold exposure. The study population was randomly selected from the entire northern region of Sweden, and contains a heterogeneous group of participants from many different backgrounds. Instead of only investigating subjects experiencing cold sensitivity as a sequela to a certain injury or disease, this study takes a general population’s perspective on cold sensitivity.

## Conclusion

Cold sensitivity was associated with both individual and external exposure factors. Being overweight was associated with a lower occurrence of cold sensitivity; and among the acquired conditions, both cold injuries, rheumatic diseases, nerve injuries, migraines, and vascular diseases were associated with the reporting of cold sensitivity. More research is needed to confirm a causal relation and determine the pathophysiological mechanisms involved. Among external exposures, cold climate and HAV exposure were associated with cold sensitivity, and both are suitable targets for primary preventive measures.
